# Clinicopathologic and endoscopic features of sessile serrated lesions and conventional adenomas: a large inpatient population-based study in China

**DOI:** 10.3389/fonc.2024.1337035

**Published:** 2024-04-04

**Authors:** Chang Tan, Geng Qin, Qian-Qian Wang, Yuan-Chen Zhou, Shu-Kun Yao

**Affiliations:** ^1^ Graduate School, Peking University China-Japan Friendship School of Clinical Medicine, Beijing, China; ^2^ Department of Gastroenterology, China-Japan Friendship Hospital, Beijing, China

**Keywords:** conventional adenoma, colorectal cancer, risk factors, inpatient, sessile serrated lesion

## Abstract

**Objectives:**

Sessile serrated lesions (SSLs) are precursors of sporadic colorectal cancer (CRC) and have distinct characteristics compared with conventional adenomas (CAs). Several lifestyle and environmental factors may play critical roles in the development of advanced lesions. Our aim is to describe the features of SSLs and CAs and further explore risk factors for advanced lesions.

**Methods:**

This is an observational study that collected demographic, endoscopic, and histological data from the China-Japan Friendship Hospital among the inpatient population with pathologically reported as SSL or CA between 2015 and 2022. We analyzed the clinicopathology and endoscopic differences between SSL alone, CA alone, and synchronous SSL+CA groups, and identified risk factors using multiple regression analysis.

**Results:**

A total of 9236 polyps from 6598 patients were included in the cohort. Patients with SSL+CA were more likely to be older (*p*=0.008), while individuals with SSL alone had a higher proportion of early-onset polyps (*p*<0.001), and SSLs were more common in advanced polyps than CAs (*p*<0.001). A greater proportion of advanced polyps in the SSL and CA groups were diagnosed as Yamada III, Yamada IV, and laterally spreading tumor (*p*=0.002, *p*<0.001, respectively), and multiple SSLs and CAs were more represented in nonadvanced polyps than in advanced polyps. In multiple regression analysis, older patients were more likely to develop advanced SSLs (aOR 1.05, 95% CI 1.02-1.09, *p*=0.005).

**Conclusion:**

SSLs and CAs have diverse demographic, endoscopic, and histological characteristics, and their advanced lesions share different risk factors, which advances the understanding of the etiology and progression of SSLs.

## Introduction

Colorectal cancer (CRC) is the third most common cancer in the world and the second leading cause of cancer death (1). In addition to the adenoma-carcinoma sequence, the serrated pathway has also gained attention in recent years, with approximately 20%-30% of sporadic CRC arising via serrated precursor lesions (2, 3). Conventional adenoma (CA) arises from truncating mutations in *APC* or other tumor suppressor genes, resulting in activation of the WNT pathway and chromosomal instability (4). However, the molecules of CRC in the serrated pathway are related to *BRAF* mutations, leading to the MAPK signaling cascade, and are also associated with microsatellite instability (MSI) and the CpG island methylator phenotype (CIMP) (5).

According to World Health Organization (WHO) criteria, serrated polyps (SPs) can be categorized into three subtypes: hyperplasic polyps (HPs), sessile serrated lesions (SSLs, previously called sessile serrated adenoma/polyp), and traditional serrated adenomas (TSAs) (6), with HPs accounting for approximately 75% and SSLs accounting for nearly 25% of SPs and TSA accounting for less than 1% (7). HPs are commonly considered benign lesions, while SSLs and TSAs, especially those with dysplasia, are precursor lesions for CRC and may contribute to interval or missed cancer development (8). SSLs usually occur in the proximal colon, are larger than 10 mm in size, and are similar in color to the surrounding mucosa, usually with a flat or sessile morphology under endoscopies (9). Previous studies have demonstrated that larger SSLs are prone to synchronous CAs, and SSLs with concurrent high-risk adenoma have an increased risk of future advanced neoplasia (10–12), which may provide evidence for guidelines recommending the interval of colonoscopy surveillance.

SSL with dysplasia (SSL-D), representing approximately 4-8% of SSLs in screening colonoscopy (7, 13), can rapidly transform into malignant lesions accompanied by marked morphological changes within several months (14). However, to the best of our knowledge, studies on SSL-D among inpatients who underwent polypectomy population are limited. Many studies have assessed lifestyle and environmental factors for SSLs and CAs, which may play important roles in their development, such as smoking status, alcohol intake, body mass index (BMI), and family history of CRC (15–17). Some risk factors are associated with both SSLs and CAs, and some are more strongly related to one type of lesion. Nevertheless, few studies have examined the risk factors for nonadvanced and advanced polyps in SSLs and CAs separately, which may provide more precise data to improve screening guidelines.

In this study, we report the characteristics of SSLs and CAs among the inpatient population scheduled for polypectomy from China-Japan Friendship Hospital. Our research aimed to describe the demographic, endoscopic, and histological features of SSL alone, CA alone, and synchronous SSL+CA groups. Furthermore, we explore risk factors and independent predictors of nonadvanced and advanced polyps in SSLs and CAs separately.

## Materials and methods

### Study design and population

This is a retrospective observational study of inpatients undergoing endoscopic polypectomy at China-Japan Friendship Hospital. Generally, patients whose largest polyp on colonoscopy is ≥6 mm should receive polypectomy in the inpatient department. All inpatients with polyps pathologically reported as SSLs or CAs between July 2015 and December 2022 were included in the study, except those who had undergone colonic surgery and those with CRC, polyposis, and inflammatory bowel disease. We collected data on demographic features including sex, age, year of endoscopy, smoking history, drinking history, BMI, and family history of CRC with the specification of a first-degree relative. If a patient had multiple polypectomies during the study period, only the first procedure was included, and all detected polyps were endoscopically removed. Because SSLs are occasionally difficult to detect, all endoscopists in this study had performed at least 500 colonoscopies independently. Ultimately, a large cohort of 9259 polyps in 6598 patients were selected and categorized into three groups based on the pathology of polyps.

### Exposure variable and outcome measures

The detailed endoscopic characteristics of polyps included polyp size, polyp location, morphology, and multiplicity. Regarding polyp locations, the most common consensus is cecum to the splenic flexure as the proximal colon, and the splenic flexure to sigmoid colon as the distal colon. For the morphology of polyps, we used the Yamada classification or laterally spreading tumor (LST) classification. From the colonoscopy cohort, we identified those with the following findings on colonoscopy: SSL alone, CA alone, and synchronous SSL+CA.

All histological diagnoses of polyps were established by two senior pathologists. Polyp histology included SSL, TSA, and CA, and the latter was further divided into tubular, villous and tubulovillous adenomas. The WHO classification recommends that histological diagnosis of SSL can be made when at least one ‘characteristic’ crypt within a lesion (18). Based on previous research (19), we considered that advanced SSL was defined as SSL ≥10 mm or with the presence of dysplasia, whereas advanced CA was defined as having any of the following features: a size equal to or greater than 10 mm, high-grade dysplasia, or tubulovillous/villous histology.

### Statistical analysis

Continuous variables are described using the mean ( ± SD), while categorical variables are presented as frequencies (proportion, %). Comparative analyses between SSL alone, CA alone, and synchronous SSL+CA groups as well as further advanced polyp subgroups were performed using Student’s *t* test, Mann−Whitney U test, one-way ANOVA, Chi-square test, and Fisher’s exact test, as appropriate. To explore the independent predictors of advanced polyps in SSLs and CAs, we performed multiple regression analysis with demographic and endoscopic variables and calculated adjusted odds ratios (aOR) and 95% confidence intervals (CI). Missing data were omitted from the analysis. All statistical analyses were performed with SPSS (version 26.0) and GraphPad Prism (version 9.4.1). A two-sided *P* value of less than 0.05 was judged as statistically significant. This study was approved by the Ethics Committee of China-Japan Friendship Hospital.

## Result

### Population and baseline characteristics

A total of 8266 inpatients underwent polypectomy with pathologically confirmed polyps between 2015 and 2022 in China-Japan Friendship Hospital. After applying exclusion criteria, the study population included 6598 individuals. Among these populations, the mean ( ± SD) age was 59.1 ( ± 11.1) years, and 64.9% of the patients were male. The mean ages of males and females were significantly different (58.4 years for males, 60.3 years for females, *p*<0.001).

Of the cohorts studied, 295 (4.5%) had SSL alone, 6201 (94.0%) had CA alone, and 102 (1.5%) had both SSL and CA. In terms of age, patients with SSL+CA were more likely to be older (61.0 ± 11.5) than those with SSL (57.2 ± 12.3) or CA (59.1 ± 11.0) alone (*p*=0.008), while individuals with SSL alone had a higher proportion of early-onset polyps than those with CA alone and SSL+CA (27.8% vs. 18.5% vs. 18.6%, *p*<0.001). Besides, those with SSL+CA were more likely to be male and had a family history of CRC than those with SSL and CA alone, but the result in this study did not reach significance. In addition, no significant differences were found in the year of endoscopy, smoking history, drinking history, or BMI between the three groups. The demographics of the patients are summarized in **Table 1**.

**Table 1:** Demographic features of patients stratified by the pathology of polyps.

	SSL alone (n=295)	CA alone (n=6201)	SSL+CA (n=102)	*P*
Sex, n (%)				0.045
Male	188 (63.7)	4017 (64.8)	78 (76.5)	
Female	107 (36.3)	2184 (35.2)	24 (23.5)	
Age (years), mean ± SD	57.2 ± 12.3	59.1 ± 11.0	61.0 ± 11.5	**0.008**
< 50	82 (27.8)	1150 (18.5)	19 (18.6)	**<0.001**
≥ 50	213 (72.2)	5051 (81.5)	83 (81.4)	
Year of endoscopy, mean ± SD	2019.5 ± 2.0	2019.3 ± 2.0	2019.1 ± 2.1	0.152
Smoking history, n (%)				0.325
No	121 (63.4)	2985 (67.9)	48 (64.0)	
Yes	70 (36.6)	1408 (32.1)	27 (36.0)	
Alcohol, n (%)				0.597
No	123 (65.8)	2944 (68.2)	48 (64.0)	
Yes	64 (34.2)	1375 (31.8)	27 (36.0)	
BMI (kg/m^2^), mean ± SD	24.5 ± 3.4	25.0 ± 3.3	24.9 ± 3.5	0.496
< 24	40 (49.4)	742 (39.1)	9 (39.1)	0.179
≥ 24	41 (50.6)	1156 (60.9)	14 (60.9)	
Family history, n (%)				0.189
No	176 (90.7)	4047 (90.4)	64 (84.2)	
Yes	18 (9.3)	429 (9.6)	12 (15.8)	

Missing data: smoking history (n=1939), alcohol (n=2017), BMI (n=4596), family history (n=1852). Bold values indicates statistically signigicant values.

### Characteristics of polyps

**Table 2** shows the endoscopic and histological characteristics of polyps. A total of 9088 polyps (148 CAs in the SSL+CA group are not included in this section) from 6598 patients, were included in this study cohort after excluding those with poorly descriptions on colonoscopy reports and those with missing pathology reports. There were 302 cases (3.3%) of SSL alone, 8677 cases (95.5%) of CA alone, and 109 cases (1.2%) of SSL+CA (focusing only on SSLs). SSL alone tended to be larger than CA alone and SSL+CA (*p*=0.045) and had a higher proportion of a polyp size ≥ 10 mm (49.3% vs. 40.9% vs. 42.2%, *p*=0.013). SSLs in SSL+CA were predominantly located in the proximal colon, while CAs were more often located in the distal colon (*p*<0.001). The proportion of low- and high-grade dysplasia in CAs was significantly higher than that in SSLs and SSL+CA (98.1% vs. 72.8% vs. 77.0%, *p*<0.001). With regard to morphology, SSLs were more highly enriched in Yamada I and LST, while CAs were more enriched in Yamada III (*p*<0.001). The proportion of multiple polyps was lower in SSLs (55.6%) than in CAs (72.5%; *p*<0.001). Among patients hospitalized for polypectomy, SSLs accounted for a greater proportion than CAs in advanced polyps (87.1% vs. 43.4%, *p*<0.001). When comparing the SSL alone with the synchronous SSL+CA group, SSLs located in the proximal colon or with dysplasia were likely to combine with CAs. As the number of TSA cases was too small, only 23 cases were included in the study cohort, and their characteristics are listed separately in **Supplementary Table 1**.

**Table 2:** Endoscopic and histological characteristics of polyps in SSL alone, CA alone and synchronous SSL+CA groups (only focus on SSLs).

	SSL alone (n=302)	CA alone (n=8677)	SSL+CA (n=109)	*P*
Size (mm), mean ± SD	10.1 ± 5.9	9.4 ± 5.2	8.9 ± 4.0	**0.045**
< 10	153 (50.7)	5130 (59.1)	63 (57.8)	**0.013**
≥ 10	149 (49.3)	3547 (40.9)	46 (42.2)	
Location, n (%)				**<0.001**
Proximal colon	144 (47.7)	3960 (45.6)	55 (50.5)	
Distal colon	100 (33.1)	3599 (41.5)	33 (30.3)	
Rectum	58 (19.2)	1118 (12.9)	21 (19.3)	
Dysplasia, n (%)				**<0.001**
Without dysplasia	82 (27.2)	161 (1.9)	25 (22.9)	
Low-grade	212 (70.2)	7960 (91.7)	82 (75.2)	
High-grade	8 (2.6)	556 (6.4)	2 (1.8)	
Morphology, n (%)				**<0.001**
Yamada I	105 (42.0)	1978 (28.0)	33 (37.5)	
Yamada II	65 (26.0)	3010 (42.6)	37 (42.0)	
Yamada III	24 (9.6)	1050 (14.9)	4 (4.5)	
Yamada IV	36 (14.4)	896 (12.7)	7 (8.0)	
LST	20 (8.0)	130 (1.8)	7 (8.0)	
Multiplicity, n (%)				**<0.001**
1	134 (44.4)	2388 (27.5)	–	
2+	168 (55.6)	6289 (72.5)	109 (100)	
Risk				**<0.001**
nonadvanced	39 (12.9)	4915 (56.6)	18 (16.5)	
advanced	263 (87.1)	3762 (43.4)	91 (83.5)	

Missing data: Morphology (n=1686). Bold values indicates statistically signigicant values.

### Nonadvanced vs. advanced polyps

In this section, we included 9236 polyps from 6598 patients for further analysis. As mentioned in the Methods section, we defined the risk of polyps by size, pathology, and degree of dysplasia. In this study cohort, there were 411 cases (4.4%) of SSLs, including 57 cases (13.9%) in the nonadvanced group, and 354 cases (86.1%) in the advanced group, and 8825 cases (95.6%) of CAs, of which 5000 cases (56.7%) were in the nonadvanced group and 3825 cases (43.3%) were in the advanced group.

As shown in **Table 3**, the locations of polyps did not differ between nonadvanced and advanced SSLs, while in the CA group, advanced polyps were more likely to be located in the distal colon and rectum (*p*<0.001). A higher proportion of advanced polyps in the SSL and CA groups were diagnosed as Yamada III, Yamada IV, and LST, while nonadvanced polyps were more enriched in Yamada I and Yamada II (*p*=0.002, *p*<0.001, respectively). Multiple SSLs and CAs were more represented in nonadvanced polyps than in advanced polyps (SSL: 80.7% vs. 65.3%, *p*=0.021; CA: 75.3% vs. 69.8%, *p*<0.001).

**Table 3:** Endoscopic and histological characteristics of nonadvanced and advanced polyps in SSLs and CAs groups.

	SSL (n=411)		CA (n=8825)	
	Nonadvanced (n=57)	Advanced (n=354)	Nonadvanced (n=5000)	Advanced (n=3825)
Size (mm), mean ± SD	6.4 ± 1.7	10.3 ± 5.7	6.3 ± 1.6	13.4 ± 5.6
	***P*<0.001**		***P*<0.001**	
Location, n (%)				
Proximal colon	32 (56.1)	167 (47.2)	2572 (51.4)	1448 (37.9)
Distal colon	16 (28.1)	117 (33.1)	1911 (38.2)	1754 (45.9)
Rectum	9 (15.8)	70 (19.8)	517 (10.3)	623 (16.3)
	*P*=0.450		***P*<0.001**	
Dysplasia, n (%)				
Without dysplasia	57 (100)	50(14.1)	144 (2.9)	17 (0.4)
Low-grade	–	294 (83.1)	4856 (97.1)	3246 (84.9)
High-grade	–	10 (2.8)	–	562 (14.7)
	***P*<0.001**		***P*<0.001**	
Morphology, n (%)				
Yamada I	24 (53.3)	114 (38.9)	1591 (40.7)	426 (13.0)
Yamada II	19 (42.2)	83 (28.3)	1973 (50.5)	1092 (33.3)
Yamada III	1 (2.2)	27 (9.2)	257 (6.6)	806 (24.6)
Yamada IV	1 (2.2)	42 (14.3)	77 (2.0)	834 (25.4)
LST	0 (0)	27 (9.2)	7 (0.2)	124 (3.8)
	***P*=0.002**		***P*<0.001**	
Multiplicity, n (%)				
1	11 (19.3)	123 (34.7)	1234 (24.7)	1154 (30.2)
2+	46 (80.7)	231 (65.3)	3766 (75.3)	2671 (69.8)
	***P*=0.021**		***P*<0.001**	

Missing data: Morphology (n=1711). Bold values indicates statistically signigicant values.

### Multivariable analysis

**Figure 1:**
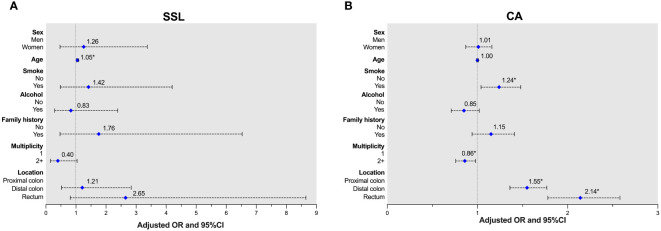
Adjusted ORs of advanced lesions in patients with either SSLs or CAs. **(A)** Age was a risk factor for SSLs. **(B)** Smoking history and polyp location were risk factors, and multiplicity was protective factor for CAs.

After univariable regression analysis, multivariate analysis was adjusted for sex, age, smoking history, drinking history, family history of CRC, multiplicity, and polyp location to analyze advanced polyps (univariable data not shown). Older patients were more likely to develop advanced SSLs (aOR 1.05, 95% CI 1.02-1.09, *p*=0.005). For advanced CAs, independent predictors were smoking history (aOR 1.24, 95% CI 1.04-1.48, *p*=0.019), multiplicity (aOR 0.86, 95% CI 0.76-0.98, *p*=0.024), and polyp location (distal colon: aOR 1.55, 95% CI 1.36-1.77, *p*<0.001; rectum: aOR 2.14, 95% CI 1.78-2.58, *p*<0.001). The associations between risk factors and advanced polyps in SSLs or CAs are shown in **Figure 1** and **Supplementary Table 2**.

## Discussion

In this large inpatient cohort undergoing polypectomy, SSLs showed distinct demographic, endoscopic and histological features compared with CAs. In addition, we explored risk factors and independent predictors of nonadvanced and advanced polyps in SSLs and CAs separately. The findings regarding SSLs among the inpatient population and their subgroups have not been previously described in published studies.

The population of this study focused on inpatients because they generally have larger and multiple polyps, and are at greater risk than the outpatient screening colonoscopy population. Our findings suggest that individuals with SSL alone had a higher proportion of patients under 50 years old than the other two groups, while patients with synchronous SSL+CA were more likely to be older. As the incidence of early-onset CRC has increased rapidly over the past two decades (20), concerns have arisen about whether SSLs pose a threat to CRC in young adults. Currently, major U.S. CRC guidelines recommend that adults aged 45 to 49 years initiate screening colonoscopy (21–23). However, the association between age and the prevalence of SSLs is unclear, with some studies indicating that the prevalence of SSLs was similar for those under and over 50 years (24, 25), which is probably due to different study populations.

For endoscopic presentation, our results are generally consistent with previous studies (3, 26). We found that compared to CAs, SSLs tended to be larger, predominantly located in the proximal colon, mostly as single lesions, mainly with a flat morphology. Besides, SSLs usually appear as indistinctive borders, cloud-like surfaces, pale lesions, mucus caps, and irregular shapes under the colonoscopy, which may help distinguish them from HPs (27, 28). However, in clinical practice, SSLs are often overlooked because of their morphology and location, as the endoscopic view of the proximal colon is often not clear enough if bowel preparation is insufficient. Therefore, the detection rate of proximal serrated polyps has been proposed as a quality indicator, associated with interval post-colonoscopy CRC (29, 30). For the histopathology findings, our data have shown that SSLs with dysplasia accounted for 74.0% of all SSLs, with a significantly higher proportion compared to those in previous research (31, 32). Upon reflection, one reason for this could be that our study was conducted at a single-center and focused only on inpatients, who typically present with relatively larger or multiple polyps. And previous studies have indicated that large size is significantly associated with SSLs with dysplasia (33), which might explain the discrepancy in results due to differences in the study population. Additionally, the diagnostic and classification criteria for SSLs have undergone multiple changes in recent years (7), which could also have an impact on these findings. Furthermore, we observed some differences in SSL alone and synchronous SSL+CA group: SSLs located in proximal colon or with dysplasia were likely to combine with CAs. Nevertheless, this finding is only a phenomenon and did not reach statistical significance. Gao et al (12) reported that individuals with proximal and large serrated polyps were more likely to have synchronous advanced neoplasia. Further studies are needed to evaluate the prognostic implications of SSLs with synchronous CAs.

Regarding risk factors and independent predictors, multiplicity surprisingly seems to be related to nonadvanced lesions. Most earlier studies have generally held that multiple adenomas are associated with advanced lesions (34, 35). Conversely, Pommergaard et al (36) found that a high number of adenomas was associated with a decreased risk of advanced adenomas. We hypothesized that multiple adenomas are attributed to genetic imbalance of cell proliferation in different individuals, the exact mechanism of which requires further exploration. In terms of polyp location, previous research has revealed that proximally located serrated polyps have higher malignant potential than distally located serrated polyps (37), while advanced CAs primarily occur in the distal colon and rectum (36). Our data have indicated that polyp location is strongly related to advanced CAs, especially those in the rectum. Existing data revealed age as a risk factor for SSLs. Anwar et al (38) reported that age ≥ 75 years was independently associated with advanced SSLs. The progression of SSLs to dysplasia and even to CRC is accelerated due to age-related methylation and synergizes with CIMP (39). Numerous studies have previously shown that smoking is associated with CAs (40–43), but it appears to be more strongly related to SSLs (15, 16), since smoking is linked with MSI-high, CIMP-positive, and BRAF mutation-positive CRC (44). However, no association between SSLs and smoking was observed in our study, possibly due to the insufficient samples and missing data. In addition, clinicians and endoscopists should counsel patients on the importance of smoking cessation.

SSLDs are rapidly progressive, difficult to detect endoscopically and commonly incompletely resected. The development of CRC through the serrated pathway implies the acquisition of a dysplastic pattern leading to SSLDs, in which MLH1 gene silencing seems to be needed (7, 45). Increasing evidence has indicated that CRC arising via the serrated pathway may be related to aberrant gastric-type mucin expression (46). As proposed by Chen and coworkers, gastric metaplasia might initiate SSL development after microbial dysbiosis (47), which potentially be the starting point for new diagnostic and therapeutic interventions.

Our study has several strengths, including a large sample cohort, detailed data collection of demography and endoscopy, confirmed polyp diagnosis with detailed recording of histopathologic information, as well as comprehensive profiling of advanced SSLs and CAs. Moreover, we compared the characteristics of patients and polyps separately in SSL alone, CA alone, and synchronous SSL+CA groups, thus providing critical insight into the etiology and features of SSLs. Furthermore, we innovatively explored multiple risk factors for advanced lesions, providing evidence for different surveillance colonoscopies. There are some limitations in our study as well. First, given the retrospective observational design, some missing or incomplete data (such as smoking history and BMI) were unavoidable, which may have contributed to biases. Second, this is a cross-sectional study, so we did not conduct follow-up and recurrence observations. Finally, this study was a large-scale single-center study, and future data from multiple centers need to be included to reduce bias. Additionally, refining the subgroups in accordance with the latest guidelines will enhance the reliability of the research findings.

## Conclusion

In summary, we focused on an inpatient population, described the demographic, endoscopic and histological profiles in SSL alone, CA alone, and synchronous SSL+CA groups, and explored multiple risk factors for advanced SSLs and CAs. Compared with CAs, SSLs tended to be larger, early-onset, predominantly located in the proximal colon, mostly as single lesions, mainly with a flat morphology, and those with dysplasia accounted for a significantly higher proportion than previously reported, which advances the understanding of the etiology and progression of SSLs. Advanced CAs were enriched in smokers and within distal colon and rectum. This study provides a detailed description of the clinicopathologic and endoscopic features of SSL, and clinicians should strengthen the understanding and attention of this lesion to avoid the risk of missing the diagnosis leading to CRC. The distinct molecular mechanisms of SSL and CA also provide new insights for targeted therapy of CRC, which is a new research direction in the future.

## Data availability statement

The raw data supporting the conclusions of this article will be made available by the authors, without undue reservation.

## Ethics statement

The studies involving humans were approved by China-Japan Friendship Hospital. The studies were conducted in accordance with the local legislation and institutional requirements. The participants provided their written informed consent to participate in this study.

## Author contributions

CT: Writing – original draft. GQ: Writing – review & editing. QW: Writing – review & editing. YZ: Writing – review & editing. SY: Writing – review & editing.
