# Supplementary material for: Clinicopathologic and endoscopic features of sessile serrated lesions and conventional adenomas: a large inpatient population-based study in China
Source: Front Oncol. 2024 Apr 4;14:1337035. doi: 10.3389/fonc.2024.1337035 (PMC11024220; doi:10.3389/fonc.2024.1337035)
Supplement: Supplementary file 1 [file Table_1.docx]

Supplementary Table 1. The clinicopathology and endoscopic characteristics of TSA

|  | **TSA (n=23)** |
| --- | --- |
| Sex, n (%) |  |
| Male | 16 (69.6) |
| Female | 7 (30.4) |
| Age (years),  mean±SD | 54.7±10.4 |
| Year of endoscopy,  mean±SD | 2020.9±1.5 |
| Smoking history, n (%) |  |
| No | 5 (41.7) |
| Yes | 7 (58.3) |
| Alcohol, n (%) |  |
| No | 4 (36.4) |
| Yes | 7 (63.6) |
| BMI (kg/m^2^),  mean±SD | 24.5±3.9 |
| Family history, n (%) |  |
| No | 9 (75.0) |
| Yes | 3 (25.0) |
| Size (mm),  mean±SD | 15±1.1 |
| Location, n (%) |  |
| Proximal colon | 9 (39.1) |
| Distal colon | 9 (39.1) |
| Rectum | 5 (21.7) |
| Dysplasia, n (%) |  |
| Without dysplasia | 7 (30.4) |
| Low-grade | 16 (69.6) |
| High-grade | 0 |
| Morphology, n (%) |  |
| Yamada I | 0 |
| Yamada II | 2 (11.1) |
| Yamada III | 5 (27.8) |
| Yamada Ⅳ | 11 (61.1) |
| LST | 0 |
| Multiplicity, n (%) |  |
| 1 | 10 (43.5) |
| 2+ | 13 (56.5) |

Missing data: smoking history (n=11), alcohol (n=12),

BMI (n=16), family history (n=11), morphology (n=5)

Supplementary Table 2. Adjusted ORs of advanced polyps in patients with SSLs or CAs

|  | **SSL** | | **CA** | |
| --- | --- | --- | --- | --- |
|  | **Adjusted OR (95% CI)** | ***P*** | **Adjusted OR (95% CI)** | ***P*** |
| Sex |  | 0.644 |  | 0.951 |
| Male | Reference |  | Reference |  |
| Female | 1.26 (0.47-3.37) |  | 1.01 (0.87-1.16) |  |
| Age | 1.05 (1.02-1.09) | **0.005** | 1.00 (0.99-1.01) | 0.185 |
| Smoking history |  | 0.523 |  | **0.019** |
| No | Reference |  | Reference |  |
| Yes | 1.42 (0.48-4.20) |  | 1.24 (1.04-1.48) |  |
| Alcohol |  | 0.731 |  | 0.077 |
| No | Reference |  | Reference |  |
| Yes | 0.83 (0.29-2.39) |  | 0.85 (0.71-1.02) |  |
| Family history |  | 0.399 |  | 0.178 |
| No | Reference |  | Reference |  |
| Yes | 1.76 (0.47-6.53) |  | 1.15 (0.94-1.41) |  |
| Multiplicity |  | 0.061 |  | **0.024** |
| 1 | Reference |  | Reference |  |
| 2+ | 0.40 (0.15-1.04) |  | 0.86 (0.76-0.98) |  |
| Location, n (%) |  |  |  |  |
| Proximal colon | Reference |  | Reference |  |
| Distal colon | 1.21 (0.52-2.84) | 0.656 | 1.55 (1.36-1.77) | **<0.001** |
| Rectum | 2.65 (0.81-8.65) | 0.106 | 2.14 (1.78-2.58) | **<0.001** |
